# Single-cell RNA sequencing identifies a subtype of FN1 + tumor-associated macrophages associated with glioma recurrence and as a biomarker for immunotherapy

**DOI:** 10.1186/s40364-024-00662-1

**Published:** 2024-10-07

**Authors:** Houshi Xu, Huihui Chai, Ming Chen, Ruize Zhu, Shan Jiang, Xiaoyu Liu, Yue Wang, Jiawen Chen, Junji Wei, Ying Mao, Zhifeng Shi

**Affiliations:** 1 Department of Neurosurgery, Peking Union Medical College Hospital, Chinese Academy of Medical Sciences and Peking Union Medical College (CAMS & PUMC), Beijing, China; 2 Research Unit of New Technologies of Micro-Endoscopy Combination in Skull Base Surgery (2018RU008), Chinese Academy of Medical Sciences and Peking Union Medical College (CAMS & PUMC), Beijing, China; 3 Department of Neurosurgery, Huashan Hospital, Shanghai Medical College, Fudan University, Shanghai, China

**Keywords:** Glioma, Recurrence, Tumor-associated macrophages, Single-cell and spatial transcriptomics, Immunotherapy

## Abstract

**Background:**

Glioma is the most common primary malignant tumor in the brain, and even with standard treatments including surgical resection, radiotherapy, and chemotherapy, the long-term survival rate of patients remains unsatisfactory. Recurrence is one of the leading causes of death in glioma patients. The molecular mechanisms underlying glioma recurrence remain unclear.

**Methods:**

Our study utilized single-cell sequencing, spatial transcriptomics, and RNA-seq data to identify a subtype of FN1 + tumor-associated macrophages (FN1 + TAMs) associated with glioma recurrence.

**Results:**

This study revealed an increased abundance of FN1 + TAMs in recurrent gliomas, indicating their potential involvement as a critical factor in glioma recurrence. A negative correlation was observed between the abundance of FN1 + TAMs in primary gliomas and the interval time to recurrence, suggesting poor prognosis for glioma patients with high levels of FN1 + TAMs. Further investigation showed that FN1 + TAMs were enriched in hypoxic tumor regions, implying that metabolic changes in tumors drive the production and recruitment of FN1 + TAMs. Additionally, FN1 + TAMs were found to contribute to the regulation of an immunosuppressive microenvironment in gliomas, and their abundance might serve as an indicator of patients’ sensitivity to immunotherapy. Finally, we developed a user-friendly website, PRIMEG (http://www.szflab.site/PRIMEG/), for exploring the immune microenvironment of primary and recurrent gliomas.

**Conclusion:**

Our findings highlight a subtype of FN1 + TAMs associated with glioma recurrence, providing new insights into potential therapeutic targets. Moreover, the abundance of FN1 + TAMs hold promise for predicting immune therapy response and aiding in more precise risk stratification of recurrent glioma patients.

**Supplementary Information:**

The online version contains supplementary material available at 10.1186/s40364-024-00662-1.

## Background

Gliomas are the most common primary malignant tumors in the cranial brain, and the standard of care (SOC) for gliomas consists of maximal surgical resection combined with postoperative radiotherapy and chemotherapy, supplemented by emerging therapies such as electric field therapy and immunotherapy, yet the overall prognosis for patients with gliomas is still unsatisfactory, with a five-year survival rate of less than 10% [1]. What makes gliomas so difficult to treat is that almost all gliomas recur after initial treatment. Compared to primary tumors, recurrent gliomas are resistant to existing therapies and have a more malignant biology [2, 3], which often means that conventional treatments will no longer benefit the patient. Therefore, it is important to identify risk factors associated with glioma recurrence, design therapeutic targets to address the key molecular mechanisms that promote recurrence, and develop treatment strategies to delay or even block recurrence [4, 5].

Glioma is an extremely heterogeneous tumor, and different molecular typing has a great impact on the prognosis of patients. The current study classifies gliomas into classic, proneuronal and mesenchymal types. Patients with mesenchymal-type gliomas have a worse prognosis and significantly shorter recurrence intervals compared to non-mesenchymal subtypes [1]. In the process of glioma development, different molecular typing will also be converted to each other [6], patients may be converted from the less malignant type of classical type to the most malignant mesenchymal type in the course of treatment. The immune microenvironment of mesenchymal gliomas is usually filled with a large number of tumor-associated myeloid cells, which constitute a unique glioma-suppressive immune ecological niche. During glioma recurrence, the molecular typing of the same patient often undergoes a switch from a non-mesenchymal subtype to a mesenchymal subtype, which is accompanied by a dynamic change in the tumor microenvironment [1], which appears to be an increase in infiltration of tumor-associated myeloid cells, suggesting that glioma recurrence events are associated with an adaptive change in the immune microenvironment.

During disease progression, the TME of recurrent gliomas differs from that of primary gliomas [7–10]. In the GLASS longitudinal glioma cohort, mesenchymal transformation of recurrent gliomas correlates with the status of macrophages in the TME, which is responsive by specific ligand-receptor interactions with tumor cells [5, 6, 11]. As the most numerous immune cells in the glioma microenvironment, macrophages play an important function in glioma development [12–15]. Macrophages (Glioma Associated Macrophages, GAMs; Tumor Associated Macrophages, TAMs) in gliomas consist of two populations: brain-resident microglia (MGs), which are derived from embryonic yolk sac precursor cells, and bone marrow-derived macrophages (BMDMs), which are sourced from circulating monocytes in the bloodstream and enriched into the tumor microenvironment by the recruitment of glioma cells in the tumor microenvironment [16–20]. Influenced by cytokines and metabolites in the tumor microenvironment, macrophages can differentiate into different types: M1 and M2. The M1/M2 classification was originally proposed based on the stimulatory response of macrophages to type 1 or type 2 cytokines in in vitro experiments [21], but it is an oversimplification. Indeed, macrophages are highly plastic and heterogeneous and can re-regulate their phenotype in response to different microenvironmental stimuli [16, 22]. Furthermore, it has been shown that M1 and M2 marker genes can be co-expressed in individual cells, implying that macrophages may present a mixed M1/M2 phenotype [16]. Recently, in addition to M1/M2 classification, high-resolution methods such as single-cell RNA sequencing (scRNA-seq) have helped to identified more complex phenotypes and diverse functional characteristics of TAMs in gliomas [23]. It has been shown that Gpnmb-overexpressing macrophages in gliomas share the Cxcl16-Cxcr6 signaling pathway and T-cell interactions with dendritic cells [24], which inhibit T-cell activation, thereby impairing T-cell killing of tumors. Recurrent glioma exhibits increased CD68 + macrophage infiltration after anti-angiogenic therapy, suggesting a potential role of TAMs in treatment resistance and tumor recurrence [12].

Here, we identified a class of TAMs subpopulations with high FN1 expression in recurrent gliomas by analyzing the changes in the glioma single-cell immune microenvironment before and after recurrence, and we found that the abundance of this class of FN1 + TAMs showed a significant negative correlation with the time interval of glioma recurrence and could serve as an independent prognostic factor for the prediction of patients’ prognosis. Combined with spatial transcriptome data, we found that FN1 + TAMs were enriched in the hypoxic regions of the tumor, suggesting that metabolic changes in the TME mediate the production and recruitment of FN1 + TAMs. We also found that FN1 + TAMs are involved in the formation of the inhibitory microenvironment of gliomas, and that FN1 + TAMs may serve as a biomarker of patient response to immunotherapy. In conclusion, our study identified a class of TAMs associated with glioma recurrence, providing new insights into potential therapeutic targets. In addition, FN1 + TAMs could serve as a promising biomarker that could contribute to more accurate risk stratification of glioma patients and prediction of response to immunotherapy. Finally, to facilitate further research and exploration of the immune microenvironment in both primary and recurrent gliomas, we have developed a user-friendly web tool, PRIMEG (Atlas of Primary and Recurrent Immune MicroEnvironment of Glioma). This resource provides comprehensive data of the immune landscape of primary and recurrent gliomas, aiming to support the development of more targeted and effective treatment approaches.

## Methods and materials

### Study design

**Fig. 1:**
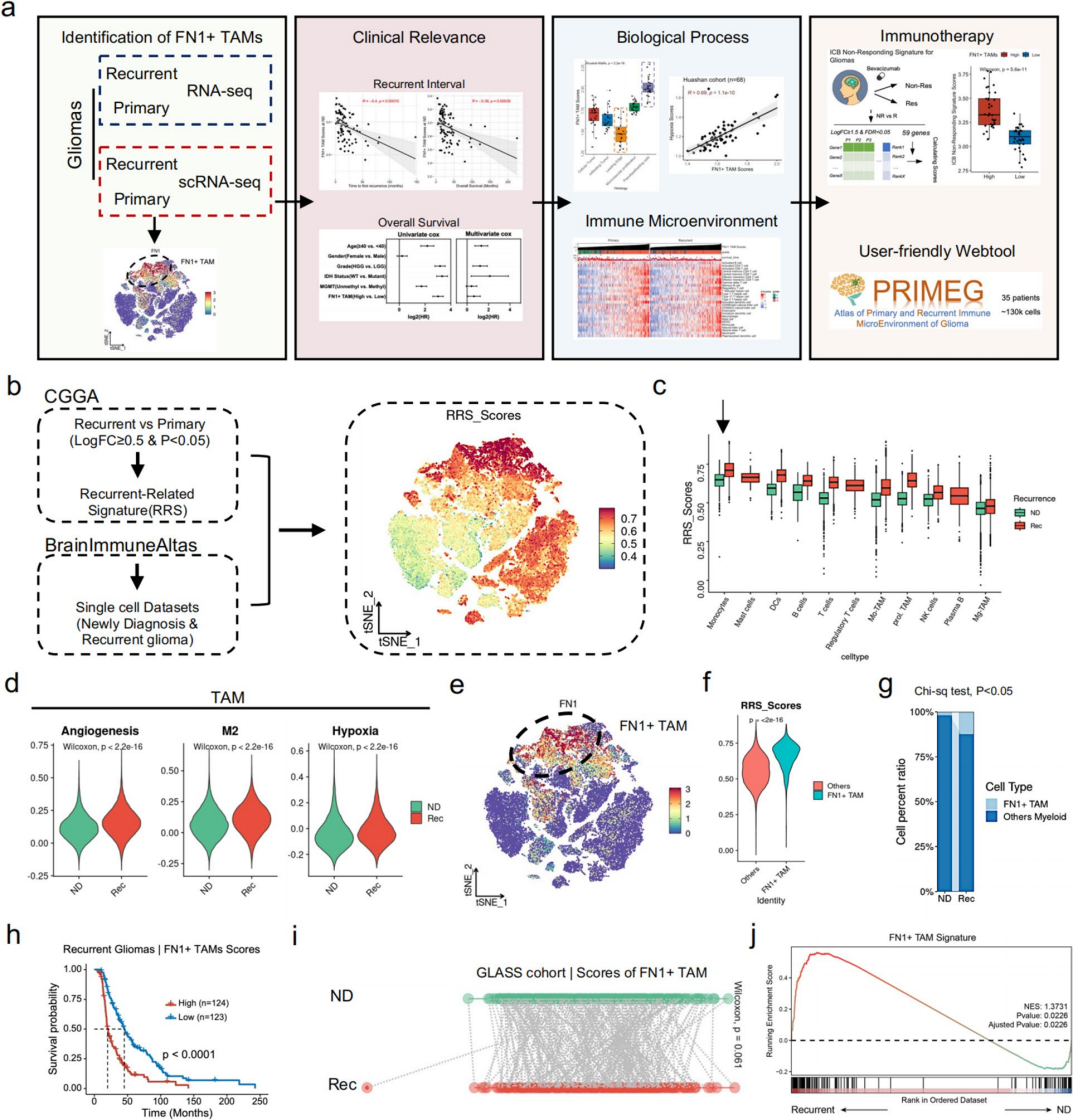
Single-cell RNA sequencing identifies a class of FN1 + TAMs associated with glioma recurrence. **a** Flowchart of the study; (**b**) Construction of RRS (Recurrent-Related Signature) and scoring of single-cell data (BrainImmuneAltas); (**c**) Distribution of RRS in different immune cells of single-cell data; (**d**) Angiogenesis, M2 macrophage, and Hypoxic gene set scores of TAMs subpopulations in primary-recurrent gliomas; (**e**) Subpopulation of TAMs with high RRS expression is characterized by high FN1 expression; (**f**) FN1 + TAMs have higher RRS scores compared to other TAMs; (**g**) The proportion of FN1 + TAMs is significantly higher in the subpopulation of recurrent glioma TAMs compared to primary gliomas; (**h**) Patients with recurrent gliomas with high FN1 + TAMs scores have a significantly poorer prognosis; (**i**) Patients with recurrent gliomas have a trend toward higher FN1 + TAMs scores compared to patients with paired primary gliomas; (**j**) GSEA showed significant upregulation of FN1 + TAMs signature in patients with recurrent glioma

The study design is presented in Fig. 1a.

### Public data sources

This study utilized multiple datasets for analysis. We used RNA-seq data of recurrent and primary gliomas from the Chinese Glioma Genome Atlas (CGGA) to calculate recurrence-related signatures (RRS), which were genes defined by log2Foldchange ≥ 0.5 and adjusted *P* value < 0.05(Fig. 1b, based on R package “limma”) [25], a lenient fold change criterion was used to include genes that may have a biologically significant but modest change in expression, which could be crucial in the context of glioma progression and immune microenvironment modulation. Since recurrent glioma is a dynamic, evolving pathological change distinct from primary glioma, and upregulated genes may reflect tumor cell growth, survival, or drug resistance, we excluded downregulated genes when defining RRS to focuses on biological changes related to glioma recurrence, specifically the activation process.

The BrainImmuneAltas (https://brainimmuneatlas.org/) single cell dataset [18]was used to compare changes in the immune microenvironment in primary and recurrent gliomas. Single cell and spatial transcriptome data of primary and recurrent glioma patients who received immunotherapy were retrieved from Mei et al.’s study [15]. Ivy Glioblastoma Atlas (https://glioblastoma.alleninstitute.org/) was used to assess the distribution of FN1 + TAMs in different glioma ecological niches [26].

For primary gliomas, gliovis [27] (http://gliovis.bioinfo.cnio.es/) was used to get the RNA-seq and clinical data of gliomas, including 1713 primary glioma patients from the CGGA (*n* = 651), TCGA (*n* = 618), and Rembrandt (*n* = 444) cohorts. For paired primary and recurrent gliomas, GLASS dataset [6] (https://www.synapse.org/glass) was used for analysis, the RNA-seq data and clinical information was obtained from synapse (syn17038081). Gene expression data of 36 glioma patients who received immunotherapy was downloaded from GSE79671 [28].

### Ethical considerations and sample collections

The Human Investigation Ethical Committee of Huashan Hospital affiliated with Fudan University deemed the use of human glioma tissues exempt from ethical review. Tumor samples were consecutively collected from the Department of Neurosurgery at Huashan Hospital between January 2020 and January 2024. The study included a total of 76 patients diagnosed with glioma, consisting of 8 with paired primary and recurrent gliomas and 68 primary gliomas. The 68 patients’ tumor samples were collected for RNA sequencing. All 68 patients underwent their first surgical procedure and had not received prior radiotherapy or chemotherapy.

## Immunohistochemical analysis

Tumor samples from patients were fixed in 4% paraformaldehyde for 24 h and subsequently embedded in paraffin wax. The paraffin blocks were then sectioned into 5-µm slices and sealed with 5% BSA overnight at 4 °C. The sections were stained with FN1 using Fibronectin/FN1 (E5H6X) Rabbit mAb #26,836 (CST) (1:100). Following PBS washes for 3 times, the sections were incubated with biotinylated anti-rabbit IgG (Vector Laboratories, CA, USA). The sections were examined using a microscope (Nikon eclipse e100), and images were processed using ImageJ software to calculate relative expression levels.

### RNA-seq raw data processing

RNA-seq reads were processed and mapped to the genome, with gene annotation performed as previously described. In brief, fastq were first assessed using FastQC v0.12.1 (FastQC, https://github.com/s-andrews/FastQC). Adapters were removed and low-quality reads filtered out using Trim Galore (TrimGalore, https://github.com/FelixKrueger/TrimGalore). Sequence alignment was conducted using hisat2 [29], with the H. sapiens_grch38_genome tar.gz file used as the reference genome. Gencode v44 annotation file (gencode.v44.annotation.gtf.gz) was utilized as the reference genome annotation.

### Single cell and spatial transcriptome data processing

Data processing and cell annotation of single-cell data of BrainImmuneAltas referred to the study of Antunes et al. [18]. We used the RRS to score primary and recurrent single cell data using AddModuleScore() function in Seurat [30] R package to determine which cells in the immune microenvironment were highly expressing RRS. Gene sets of angiogenesis, M2 macrophage, and hypoxia pathways were obtained from the study of Wang et al. [31]. Single cell data of primary and recurrent glioma patients was scored with FN1 + TAMs signatures using AddModuleScore(). 10x genomics spatial transcriptome data of primary and recurrent glioma patients who received immunotherapy were utilized for further analysis [15]. We imported the spatial transcriptome data into the Seurat package for analysis and used the AddModuleScore() function to score the expression of the gene module of interest on each spot, thereby assessing the spatial expression patterns of specific gene modules. To infers vectors of developmental directions for the TAMs, we used RNA velocity analysis [32] and Sligshot [33] to perform pseduotime analysis. We used the RunKNNPredict() function of the SCP (https://github.com/zhanghao-njmu/SCP) R package for cell label transferring with default parameters. Shiny (https://github.com/rstudio/shiny) and Cirrocumulus (https://github.com/lilab-bcb/cirrocumulus) were used for the construction of the single cell website. The corrected Harmony embeddings were utilized instead of principal components for unsupervised clustering of cells, employing graph-based clustering based on SNN as implemented in the Seurat v.4.3.1 R package (with default parameters). The clustering results were visualized in two-dimensional scatter plots (via TSNE) using both the Seurat v.4.3.1 and SCP (https://github.com/zhanghao-njmu/SCP) R packages.

### Bioinformatic analysis

In processing of the Ivy Glioblastoma Atlas, we focused on comparing the transcriptome differences between the pseudopalisading cell region (PC region) and the leading edge region (LE region) and analyzed Hallmark 50 features using the GSVA algorithm using R package “GSVA” [34]. Differentially expressed genes (DEGs) between PC and LE region were analyzed by R package “limma” [25], we applied more stringent criteria (log2Foldchange ≥ 1.5 and adjust *P*-value < 0.05) to focus on the most significant changes. The Gene Ontology Database (GO) was used to annotate the biological functions with the R package “clusterProfiler” [35].

For GLASS, CGGA, TCGA, Rembrandt and Huashan cohorts, single sample gene set enrichment analysis (ssGSEA) was employed to compute enrichment scores, reflecting the absolute enrichment level of a gene set in each sample within each dataset, using the R package “GSVA” [34]. Normalized enrichment scores were calculated for each signature. Gene set signatures for 28 types of immune cells were obtained from a prior study [36]. MCPcounter analysis [37] (https://github.com/ebecht/MCPcounter) was performed to estimate the population abundance of tissue-infiltrating immune and stromal cell populations. To investigate the relationship between FN1 + TAMs scores and immune status, 25 immune-related gene sets from previous studies were obtained [38], encompassing both innate and adaptive immune responses. The cancer immunity cycle gene sets were obtained from TIP [39] (http://biocc.hrbmu.edu.cn/TIP). The subclass mapping (SubMap) [40] method was used to evaluate the correspondence of the two subgroups and the patients with different immunotherapy responses.

### Statistical analysis

Survival (V3.5-8, https://cran.r-project.org/web/packages/survival/) and survminer (V0.4.9, https://cran.r-project.org/web/packages/survminer) R packages were used to assess the correlation between FN1 + TAMs subgroups and overall survival. All statistical analyses were conducted using R software version 4.2.0. To compare differences between two groups, either Student’s t-test or Wilcoxon test was employed. Correlations between variables were evaluated using Pearson correlation coefficients. A difference in the mean was considered statistically significant if the *P*-value was less than 0.05. The levels of statistical significance were indicated as follows: *P < 0.05; **P < 0.01; ***P < 0.001; ****P < 0.0001.

## Results

### Single-cell RNA sequencing identifies FN1 + TAMs associated with glioma recurrence

To investigate the alterations in immune microenvironment cells in primary and recurrent gliomas, we initially analyzed RNA-seq data from the Chinese Glioma Genome Atlas (CGGA) to identify up-regulated genes in recurrent gliomas. We defined recurrence-related signatures (RRS) as genes exhibiting a log2Foldchange ≥ 0.5 and *p*-value < 0.05. Utilizing these RRS, we scored single-cell data from the BrainImmuneAtlas for both recurrent and primary gliomas (Fig. 1b) to pinpoint immune cells expressing high levels RSS scores (see Methods). Our findings indicated that RRS expression was highest in monocytes, with recurrent gliomas showing elevated RRS expression in monocytes compared to primary gliomas (Fig. 1c).

We then narrowed our focus to tumor-associated macrophages (TAMs). By comparing the angiogenesis, M2, and hypoxia gene sets in TAMs from primary and recurrent gliomas, we observed significant signaling up-regulations in TAMs within recurrent gliomas (Fig. 1d). Further, we analyzed differentially expressed genes in TAMs with high RRS compared to other TAMs (Supplementary Material 1), revealing that high RRS TAMs were characterized by elevated FN1 expression (Fig. 1e).

The FN1 gene, located on chromosome 2q35, encodes fibronectin, a protein integral to the extracellular matrix and involved in processes such as cell adhesion, migration, and wound healing. FN1 is expressed by various cells, including some tumor cells [41], fibroblasts [42], macrophages [43, 44], and endothelial cells [45]. Macrophages with high FN1 expression exhibit a stronger inflammatory response and may contribute to tumor invasion and progression [42, 44, 46]. We hypothesized that FN1 + TAMs might be involved in glioma recurrence.

Subsequent comparisons between FN1 + TAMs and other TAMs showed significant overexpression of RRS in FN1 + TAMs (Fig. 1f). The proportion of FN1 + TAMs was notably higher in recurrent gliomas than in primary gliomas (Fig. 1g), suggesting a pivotal role for FN1 + TAMs in glioma recurrence. Prognostic analysis revealed that patients with recurrent gliomas and high levels of FN1 + TAMs had significantly poorer outcomes (Fig. 1h). Analyzing changes in FN1 + TAM signatures between primary and recurrent gliomas, we found a trend towards upregulation in recurrent cases (Fig. 1i, *p* = 0.061). Gene Set Enrichment Analysis (GSEA) further confirmed significant upregulation of FN1 + TAM signatures in recurrent gliomas (Fig. 1j). To further elucidate the dynamics of macrophage populations in gliomas, we have conducted RNA velocity analysis on single-cell RNA-seq data. In order to verify the reliability of the results, we simultaneously used Slingslot [33] for the pseduotime analysis of TAMs. Preliminary results from the velocity analysis indicate that FN1 + TAMs exhibit distinct transition patterns (Supplementary Fig. 1), supporting our hypothesis that they play a unique role in glioma recurrence.

In summary, our study identified a subset of TAMs with high FN1 expression through single-cell RNA sequencing. These FN1 + TAMs are significantly enriched in recurrent gliomas, differ markedly from TAMs in primary gliomas, and are closely associated with the prognosis of patients with recurrent gliomas.

### FN1 + TAMs scores negatively correlate with glioma recurrence intervals

**Fig. 2:**
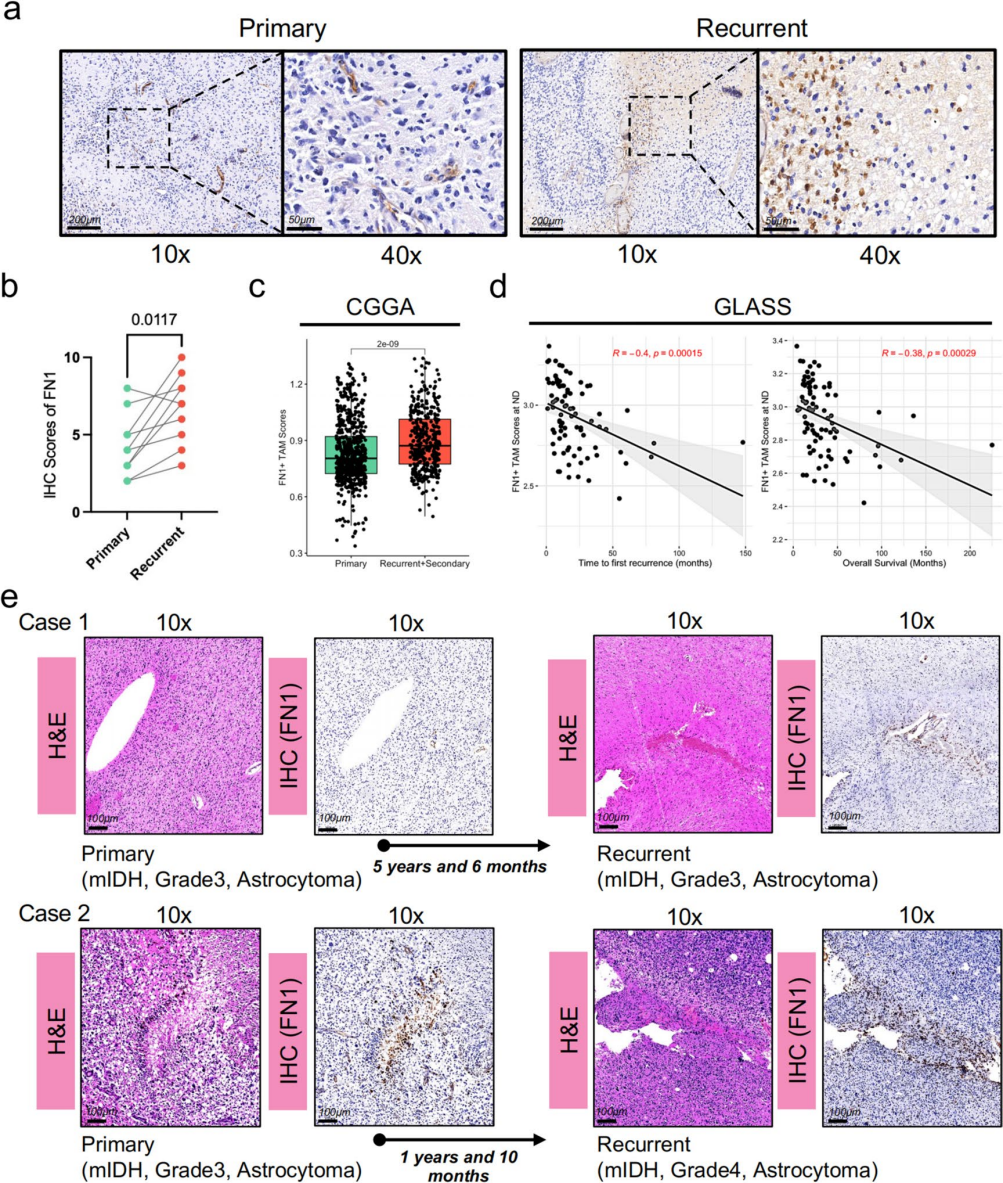
FN1 + TAMs scores significantly correlate with glioma recurrence interval (**a**, **b**) Immunohistochemical analysis showed significantly higher FN1 levels in recurrent gliomas compared to primary gliomas; (**c**) CGGA database also demonstrated significantly higher FN1 + TAMs scores in recurrent gliomas compared to primary gliomas; (**d**) FN1 + TAMs scores were significantly negatively correlated with recurrence intervals and overall survival time; (**e**) Two primary glioma patients with different levels of FN1 exhibited remarkably different recurrence intervals. Scale bar:100 μm

To further investigate FN1 expression in primary and recurrent gliomas, we conducted immunohistochemical analysis on eight paired glioma samples obtained from Huashan Hospital. The analysis revealed that FN1 expression was significantly elevated in recurrent gliomas compared to primary gliomas (Fig. 2a, b). Consistently, analysis of the CCGA database showed a significant increase in FN1 + TAMs scores in recurrent gliomas compared to primary gliomas (Fig. 2c).

A recent single-center study of recurrent gliomas showed that the interval between glioma recurrences was strongly associated with overall prognosis, and that patients with gliomas with short recurrence intervals require closer follow-up. We hypothesized that the abundance of FN1 + TAMs might correlate with the interval times. To test this, we examined the correlation between FN1 + TAM scores and glioma recurrence intervals using data from the GLASS cohort [6]. Our analysis demonstrated a significant negative correlation between FN1 + TAM scores in primary gliomas and both recurrence intervals and overall survival times (*R* = -0.4 and *R* = -0.38, respectively; Fig. 2d). These findings suggest that FN1 + TAM scores could serve as a biological marker for predicting recurrence and prognosis in gliomas.

For external validation, we selected two in eight paired glioma patients with markedly different recurrence intervals (Fig. 2e). The results indicated that glioma with high FN1 expression (Case 2) had significantly shorter recurrence intervals compared to gliomas with low FN1 expression (Case 1), with intervals of 1 year and 10 months versus 5 years and 6 months, respectively. These findings support the potential role of FN1 + TAMs as predictors of recurrence intervals in gliomas, newly diagnosis glioma patients with high FN1 + TAMs scores may need a closer follow-up.

### FN1 + TAMs scores correlate with glioma malignancy and is an independent risk factor for primary gliomas

**Fig. 3:**
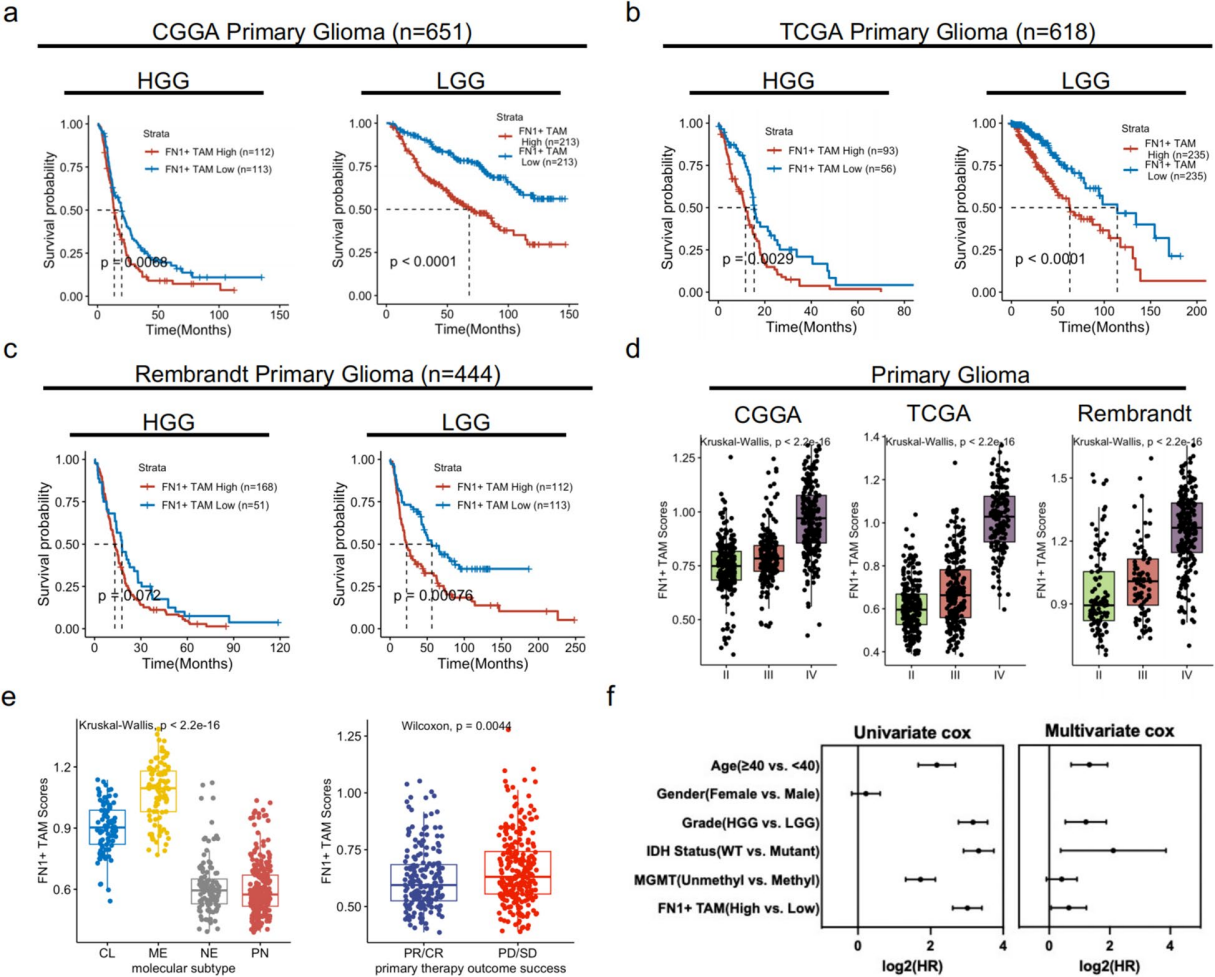
Prognostic significance of FN1 + TAMs in primary glioma patients.  **a**-**c** Kaplan-Meier survival analysis illustrating overall survival in primary glioma patients stratified by FN1 + TAMs scores. High FN1 + TAMs scores are associated with significantly lower overall survival compared to low FN1 + TAMs scores (CGGA: *n* = 651, TCGA: *n* = 618, Rembrandt: *n* = 444); **d** Association of FN1 + TAMs scores with glioma grade, indicating a significant increase in FN1 + TAMs scores with higher grade; (**e**) Distribution of FN1 + TAMs scores across molecular classifications of gliomas, highlighting mesenchymal-like gliomas with the highest FN1 + TAMs scores; (**f**) Univariate and multivariate Cox regression analyses demonstrating FN1 + TAMs scores as an independent prognostic factor for primary glioma patients, adjusted for age, gender, grade, IDH mutation status, and MGMT promoter methylation status

The aforementioned findings indicate that FN1 + TAMs play a critical role in glioma recurrence. To further explore their prognostic value in primary glioma patients, we conducted a survival analysis including 1713 primary glioma patients from the CGGA (*n* = 651), TCGA (*n* = 618), and Rembrandt (*n* = 444) databases. The results demonstrated that patients with high FN1 + TAMs scores had significantly lower overall survival compared to those with low FN1 + TAMs scores (Fig. 3a, b, and c).

Additionally, FN1 + TAMs scores were observed to increase significantly with primary glioma grade, suggesting that FN1 + TAMs scores reflect the malignancy of gliomas. Analysis of FN1 + TAMs scores across different molecular classifications of gliomas revealed that mesenchymal-like gliomas exhibited the highest FN1 + TAMs scores (Fig. 3e). Furthermore, patients with progressive disease/stable disease (PD/SD) had higher FN1 + TAMs scores compared to those with partial response/complete response (PR/CR), indicating that FN1 + TAMs scores may predict patient response to treatment (Fig. 3e).

To determine whether FN1 + TAMs scores could serve as an independent risk factor for primary glioma, we performed univariate and multivariate cox analyses, accounting for age, gender, grade, IDH mutation status, MGMT promoter methylation status, and FN1 + TAMs scores. The results confirmed that FN1 + TAMs scores retained its prognostic impact even when adjusted for multiple prognostic factors (Fig. 3f), suggesting that FN1 + TAMs scores could be utilized as an independent risk factor for primary gliomas.

### FN1 + TAMs are predominantly enriched in the pseudopalisading cell (PC) regions of gliomas and exhibit a strong association with hypoxia signals

**Fig. 4:**
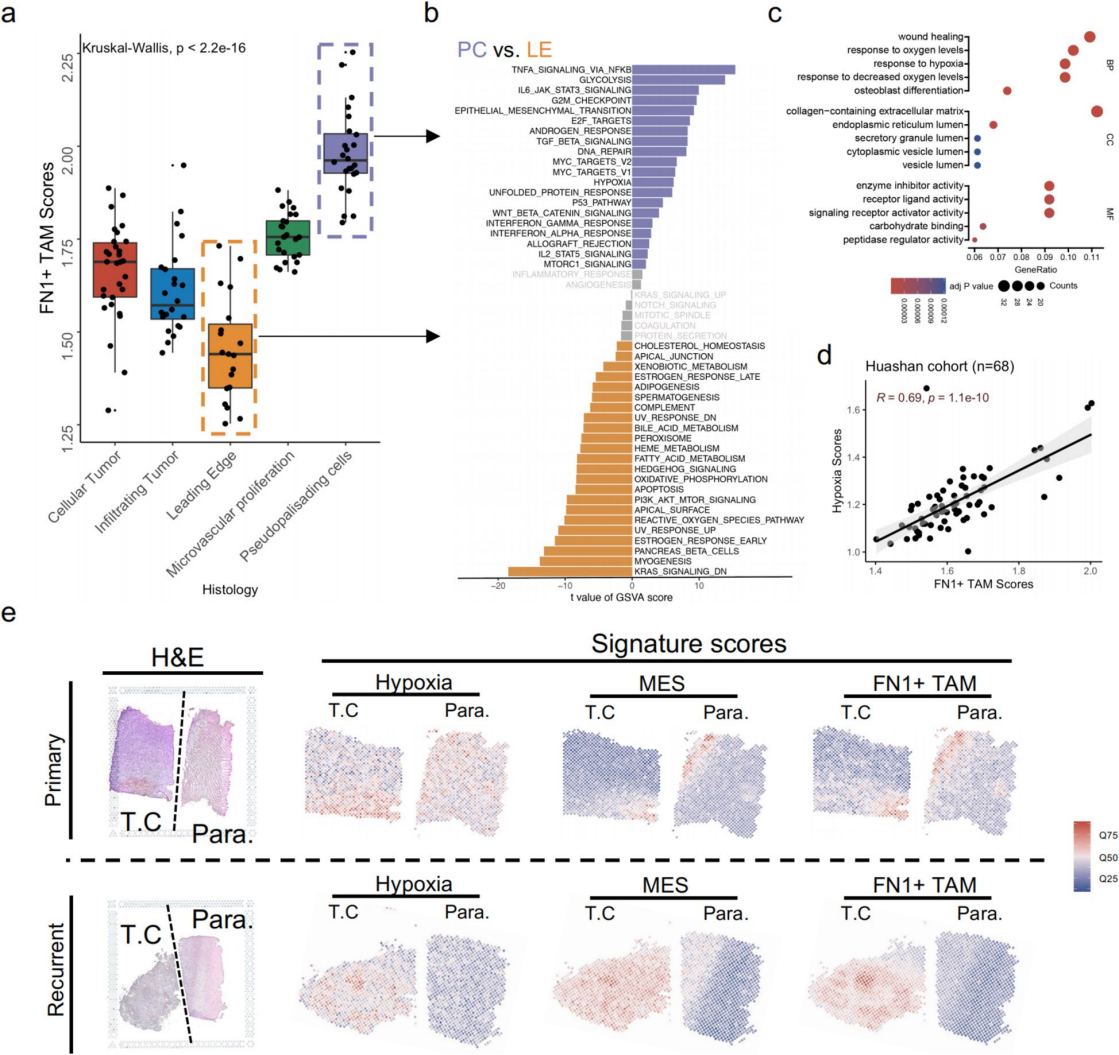
Spatial distribution and metabolic association of FN1 + TAMs in gliomas (a) Evaluation of FN1 + TAMs scores across glioma regions using bulk RNA-seq data from the Ivy Glioblastoma Atlas, showing highest scores in pseudopalisading cell (PC) regions and lowest in the leading edge (LE); (b) GSVA analysis of 50 Hallmark gene sets comparing PC and LE regions, highlighting elevated immune-related signals (TNF-a via NFkB, IL6/JAK/STAT3, TGF beta) and hypoxia signaling in the PC region; (c) GO enrichment analysis of upregulated genes in the PC region; (d) Correlation between FN1 + TAM scores and hypoxia scores in 68 gliomas from Huashan Hospital, indicating a significant association between FN1 + TAMs and hypoxic conditions; (e) Spatial transcriptomic data analysis showing co-localization of FN1 + TAMs with hypoxia and mesenchymal signals in recurrent gliomas

Gliomas are highly heterogeneous malignant tumors, with variability not only between patients but also within different regions of the same tumor [13]. To investigate the spatial distribution of FN1 + TAMs, we evaluated bulk RNA-seq data from the glioma niche [26](Ivy Glioblastoma Atlas) (Fig. 4a). The results showed that FN1 + TAMs scores were highest in the pseudopalisading cell (PC) region and lowest in the glioma leading edge (LE). We speculated that this distribution might be closely related to the microenvironmental characteristics of different regions. Comparing the transcriptome differences between the PC and LE regions, we analyzed the Hallmark 50 features using the GSVA algorithm (Fig. 4b). The PC region predominantly exhibited high expression of immune-related signals, including TNF-a signaling via NFkB, IL6/JAK/STAT3, and TGF beta signaling. Additionally, hypoxia signaling was significantly up-regulated in the PC region compared to the LE region, suggesting an altered metabolic ecology in the PC region.

Further GO enrichment analysis of the upregulated genes in the PC region revealed that their biological functions were mainly focused on wound healing, response to oxygen levels, and response to hypoxia, indicating an upregulation of hypoxic signaling (Fig. 4c). Recent studies have shown that the hypoxia ecological niche plays a crucial role in glioma development [13, 47]. Hypoxia can up-regulate the transcription factor HIF1A in TAMs, which activates the downstream LGMN/GSK-3b/STAT3 signaling pathway, promoting the inhibitory effect of TAMs on CD8 T cells and creating an immune-suppressive microenvironment conducive to glioma growth [47]. We hypothesized that the enrichment of FN1 + TAMs in the PC region was associated with the up-regulation of hypoxia-related signaling pathways. Our analysis of RNA-seq data from 68 gliomas at Huashan Hospital revealed a significant positive correlation between FN1 + TAM scores and hypoxia scores (*R* = 0.69, Fig. 4d), suggesting that FN1 + TAMs are linked to hypoxia and are significantly enriched in hypoxic niches.

Subsequent analysis of the distribution of hypoxia signals, mesenchymal signals, and FN1 + TAMs scores in the microenvironment using spatial transcriptomic data corroborated these findings. We observed that recurrent gliomas exhibited significant up-regulation of FN1 + TAMs scores and mesenchymal signals, with a clear spatial co-localization of FN1 + TAMs with hypoxia signals (Fig. 4e). Collectively, these results indicate that FN1 + TAMs show heterogeneous spatial distribution in tumors, are mainly associated with hypoxia signals, and highlight the role of the glioma metabolic microenvironment in regulating FN1 + TAMs.

### The abundance of FN1 + TAMs is strongly associated with an immune-suppressive microenvironment in gliomas

**Fig. 5:**
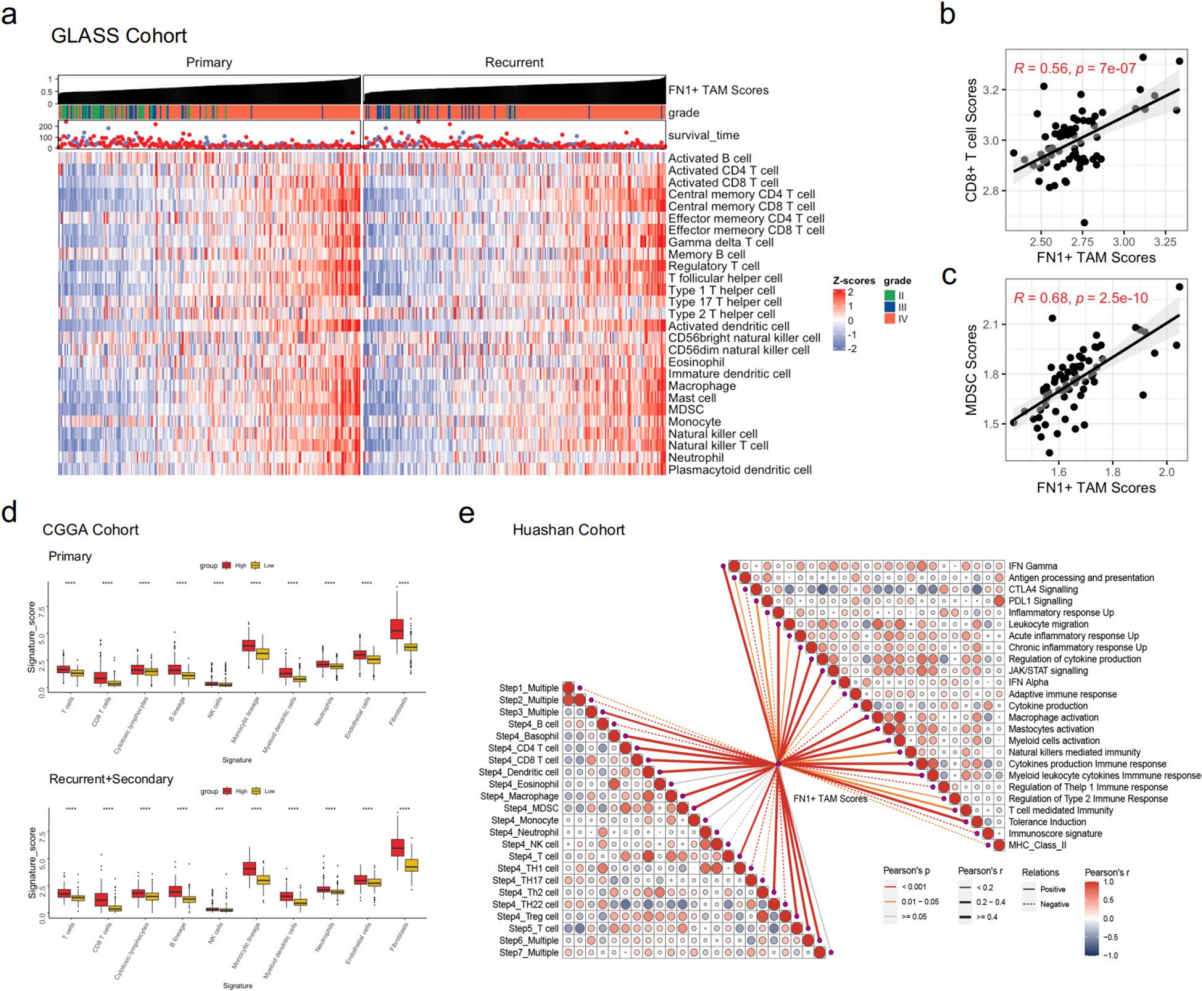
The role of FN1 + TAMs in the glioma immune microenvironment (**a**) Correlation analysis using ssGSEA shows significant positive correlations between FN1 + TAMs scores and T cells and MDSCs infiltration in primary and recurrent gliomas; (**b**, **c**) Validation in the Huashan cohort confirms the close link between FN1 + TAMs and immune cell infiltrations; (**d**) Analysis using the MCPcounter algorithm in the CGGA cohort reveals higher abundances of T cells, monocytes, myeloid cells, and fibroblasts in gliomas with high FN1 + TAMs scores; (**e**, lower left) ssGSEA analysis of the tumor immune cycle steps in the Huashan cohort shows positive correlations of FN1 + TAMs scores with steps 3 (triggering and activation), 4 (immune cells transport to the tumor), and 6 (T cell recognition of the tumor), but no significant correlation with step 7 (tumor killing); (e, upper right) Analysis of immune-related pathways demonstrates significant positive correlations between FN1 + TAMs scores and pathways like IFN Gamma, PDL1 signaling, leukocyte migration, and chronic inflammatory response UP, highlighting immune pathway activation and T cell-mediated immune surveillance evasion in gliomas

TAMs play a crucial role in glioma development, promoting tumor cell proliferation and metastasis while inhibiting CTL and NK cell activity in the tumor microenvironment [48–50]. This suppression reduces the body’s immune response to the tumor, facilitating immune evasion. To investigate the relationship between FN1 + TAMs and the glioma immune microenvironment, we analyzed the correlation between FN1 + TAMs scores and levels of immune cell infiltration using the ssGSEA algorithm to quantify 28 immune cell types. The results revealed a significant positive correlation between FN1 + TAMs and T cells and MDSCs in both primary and recurrent gliomas (Fig. 5a). This finding was validated in the Huashan cohort (Fig. 5b, c), indicating that FN1 + TAMs are closely linked to microenvironmental regulation.

FN1 + TAMs were associated with both “hot” and “cold” tumor characteristics, suggesting a unique microenvironmental composition in gliomas. Further analysis using the MCPcounter algorithm in the CGGA cohort showed that primary and recurrent gliomas with high FN1 + TAMs scores had significantly higher abundances of T cells, monocytes, myeloid cells, and fibroblasts compared to those with low scores (Fig. 5d). This suggests that FN1 + TAMs are integral to the glioma microenvironment, with high FN1 + TAMs indicating both “cold” and “hot” microenvironmental features.

We also examined the relationship between FN1 + TAMs abundance and tumor immunity, focusing on immune-related pathways. The tumor immune cycle, which describes the generation of anticancer immune responses in seven steps, was analyzed using the ssGSEA algorithm on RNA-seq data from Huashan cohort. Most steps of the cycle were positively correlated with FN1 + TAMs scores, including triggering and activation (step 3), immune cells transport to the tumor (step 4), and T cell recognition of the tumor (step 6). These positive correlations revealed effector TIIC infiltration in the glioma immune microenvironment (Fig. 5e, lower left). However, no significant correlation was found between FN1 + TAMs scores and tumor killing (step 7), suggesting dysregulation in the immune microenvironment for glioma cell killing in patients with high FN1 + TAMs scores.

Glioma has a suppressive immune microenvironment contributing to immunotherapy resistance [15, 16]. We quantified the immune phenotype of gliomas using a set of genes associated with adaptive and innate immune responses. The results showed that FN1 + TAMs scores were significantly positively correlated with signaling pathways such as IFN Gamma, PDL1 signaling, leukocyte migration, and chronic inflammatory response UP, indicating significant immune-related pathway activation in glioma patients with high FN1 + TAMs scores. This activation is closely linked to T cell-mediated immune surveillance evasion in gliomas (Fig. 5e, upper right).

These findings suggest that high FN1 + TAMs scores reflect T cell dysfunction in glioma patients. T cell function is critical for effective immunotherapy [51–53], as their activity and functional status directly impact their ability to recognize and kill tumors, thereby influencing immunotherapy efficacy.

### The abundance of FN1 + TAMs predicts glioma patients’ response to immunotherapy

**Fig. 6:**
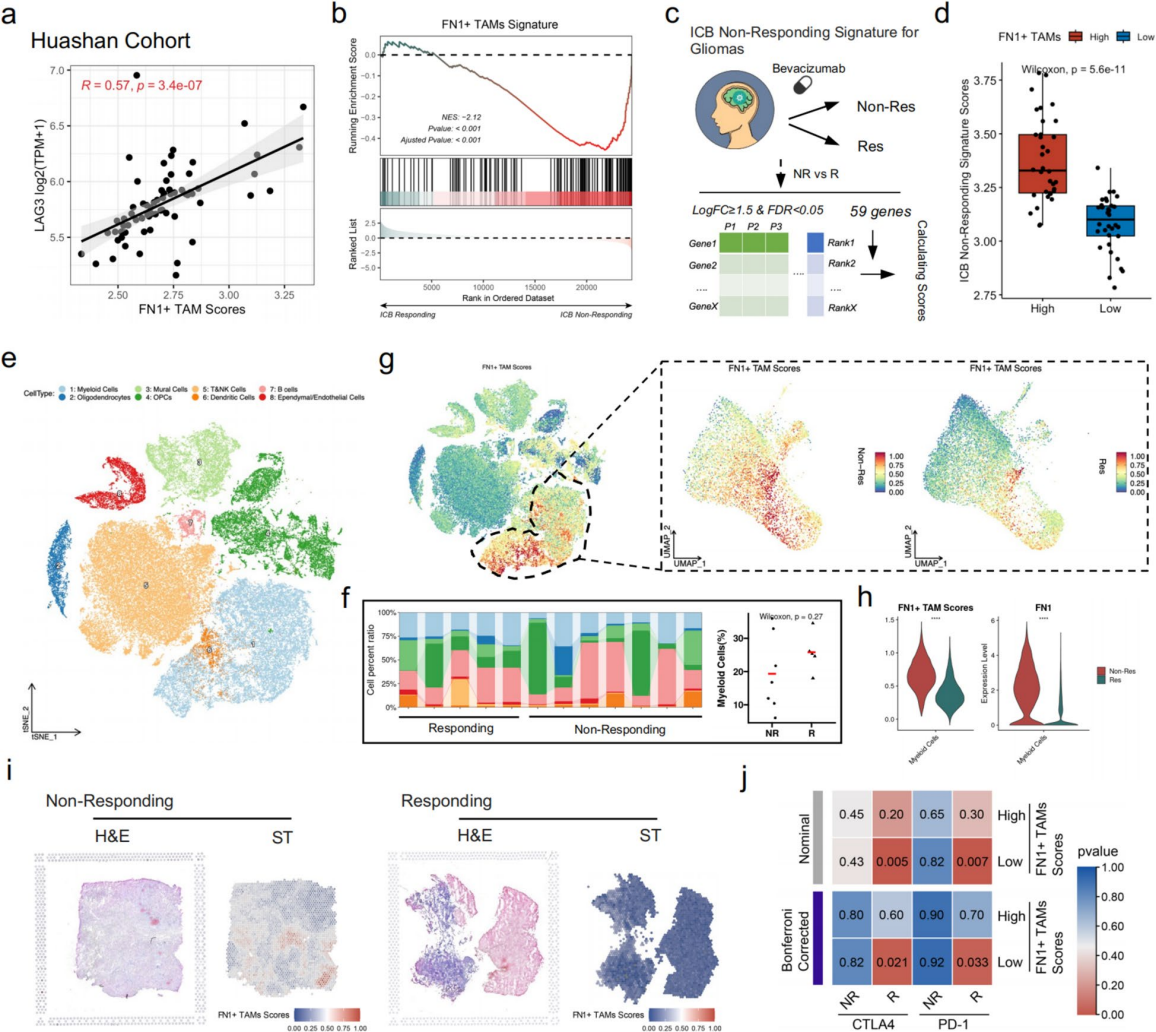
FN1 + TAMs as predictors of glioma patients’ response to immunotherapy. **a** Significant positive correlation between FN1 + TAMs scores and T cell exhaustion marker LAG3 expression; (**b**) GSEA of FN1 + TAMs signature between immunotherapy responders and non-responders shows significant upregulation in non-responders; (**c**) Construction of a glioma immunotherapy non-response signature based on differential genes between responders(R) and non-responders (NR) to bevacizumab, (**d**) Non-response scores significantly higher in the high FN1 + TAMs scores group in the Huashan cohort; (**e**-**h**) Single-cell data analysis of immunotherapy-treated glioma patients reveals FN1 + TAMs scores and FN1 expression in myeloid cells are significantly higher in the NR group compared to the R group; (**i**) Spatial transcriptome data confirmed lower FN1 + TAMs scores in the R group than in the NR group; (**j**) SubMap analysis indicates higher sensitivity to anti-PD1 and anti-CTLA4 treatments in the low FN1 + TAMs subgroup

We then investigated the correlation between FN1 + TAMs scores and T cell exhaustion marker, finding a significant positive correlation with LAG3 expression (Fig. 6a). This suggests a dysfunctional T cell microenvironment in patients with high FN1 + TAMs expression. We then compared the FN1 + TAMs signature between patients who responded to immunotherapy and those who did not in an immunotherapy glioma cohort. The results showed significant upregulation of FN1 + TAMs signature in non-responders (Fig. 6b), indicating that FN1 + TAMs abundance may reflect glioma patients’ response to immunotherapy.

Using this cohort, we constructed a glioma immunotherapy non-response signature (Fig. 6c). Briefly, glioma patients were divided into response (R) and non-response (NR) groups based on their response to bevacizumab. Differential genes between NR and R groups were identified, with logFoldchange ≥ 1.5 and FDR < 0.05 defining the glioma immunotherapy non-response signature genes. Patients in the Huashan cohort were scored using this signature, revealing that the non-response scores were significantly higher in the high FN1 + TAMs scores group (Fig. 6d), further suggesting FN1 + TAMs as a predictive biomarker for glioma immunotherapy response.

To validate these results, we analyzed single-cell data from immunotherapy-treated glioma patients [15]. This dataset included TME cells from recurrent gliomas with immunotherapy non-responding and responding, classified into eight major cell populations (Fig. 6e). Comparing myeloid cell abundance between NR and R groups showed no significant difference (Fig. 6f). However, FN1 + TAMs scores were significantly higher in the myeloid cells of NR group (Fig. 6g), and FN1 expression in myeloid cells was elevated compared to the R group (Fig. 6h). Spatial transcriptome data further confirmed that FN1 + TAMs scores were lower in the R group than in the NR group (Fig. 6i). These findings demonstrate that FN1 + TAMs, associated with glioma recurrence, are also linked to immunotherapy sensitivity, explaining the poor response of recurrent gliomas to immunotherapy.

Additionally, the SubMap algorithm [40] compared expression profiles between FN1 + TAMs subgroups in glioma patients and 47 melanoma patients with immunotherapy. It revealed that patients in the low FN1 + TAMs subgroup were more sensitive to anti-PD1 and anti-CTLA4 treatments (*P*-value for Bonferroni correction = 0.033 and 0.021) (Fig. 6j), consistent with previous findings.

In summary, FN1 + TAMs may be a valuable indicator for quantifying the tumor immune microenvironment and predicting glioma patients’ response to immunotherapy.

### Construction of a user-friendly website for exploring primary and recurrent immune microenvironment of glioma

Increasing evidence suggests that gliomas undergo profound changes in the immune microenvironment during recurrence [10, 54, 55]. These changes are reflected not only in cell proportions but also in significant alterations in cell subpopulations and functions. To facilitate in-depth research on the immune microenvironment of recurrent gliomas, we have developed a comprehensive single-cell immune microenvironment database for primary and recurrent gliomas (Atlas of Primary and Recurrent Immune MicroEnvironment of Glioma, PRIMEG).

**Fig. 7:**
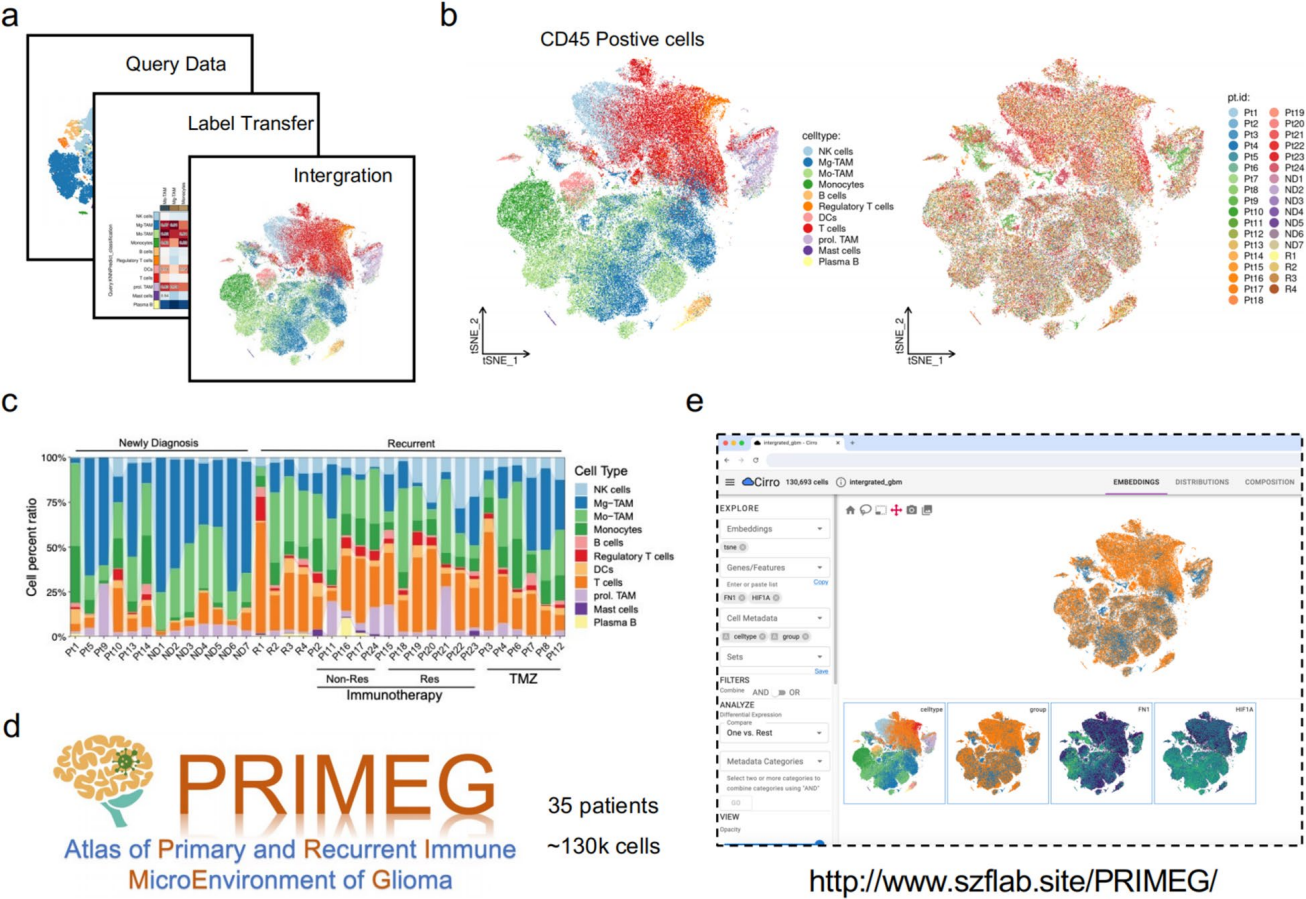
Development of the PRIMEG webtool for primary and recurrent glioma immune microenvironment research. **a** Schematic overview of the database construction process, including the use of BrainImmuneAtlas as a reference dataset, annotation of single-cell data using transfer learning, and integration/batch correction of datasets; (**b**-**d**) The combined dataset includes ~130,000 high-quality immune cells from 35 glioma patients, consisting of 13 primary and 22 recurrent gliomas, with 12 of the recurrent cases having received immunotherapy, PRIMEG is accessible through an online platform with a user-friendly interface, allowing users to perform gene expression analysis and subpopulation annotation by simply clicking on the navigation bar; (**e**) Screenshots of the webtool interface, demonstrating the ease of use for researchers to explore and analyze the immune microenvironment of primary and recurrent gliomas

To construct this database, we first used the BrainImmuneAtlas as a reference dataset. Given that our focus is on immune cells, we excluded non-immune cell components from the single cell dataset [15]. We then annotated the processed single cell data using transfer learning and integrated and batch-corrected the two datasets with Harmony software(Fig. 7a, b). The resulting combined dataset includes approximately 130,000 high-quality immune cells from 35 glioma patients, comprising 13 primary and 22 recurrent gliomas, with 12 of the recurrent cases having received immunotherapy (Fig. 7b, c).

This database is accessible via an online platform with a user-friendly interface (Fig. 7d, e), enabling researchers to easily perform tasks such as gene expression analysis and subpopulation annotation by just clicking the navigation bar on the left and top of the webtool.

We have established the first single-cell database focused on the immune microenvironment of primary and recurrent gliomas, PRIMEG (http://www.szflab.site/PRIMEG/), which we believe will significantly advance researches into the molecular mechanisms and therapeutic targets of recurrent gliomas.

## Discussion

With the development of high-throughput sequencing technology, we have a better understanding of the progression of gliomas. In this study, we have identified a distinctive FN1 + tumor-associated macrophages (TAMs) population that is significantly associated with glioma recurrence by single-cell RNA sequencing. The elevated expression of FN1 in recurrent gliomas, as verified through both immunohistochemical analysis of paired glioma samples and database comparisons, underscores the importance of FN1 + TAMs in glioma pathophysiology. The significant negative correlation between FN1 + TAMs score and recurrence interval and overall survival suggests that FN1 + TAMs may serve as a predictive biomarker for recurrent glioma events and prognosis. Specifically, the notable differences in prognosis between primary glioma patients with high and low FN1 + TAMs scores further support the prognostic value of FN1 + TAMs in primary gliomas.

Additionally, our findings indicate that FN1 + TAMs play a pivotal role in shaping the immune microenvironment of gliomas. The upregulation of multiple steps in the tumor immune cycle, particularly those associated with immune cell recruitment and T cell recognition, highlights the complex interaction between FN1 + TAMs and tumor immunity. This enhanced activity could contribute to increased infiltration of effector tumor-infiltrating immune cells (TIICs) in the glioma immune microenvironment, potentially influencing the tumor’s response to treatment.

Interestingly, the analysis of immunotherapy-treated glioma RNA-seq data revealed that FN1 + TAMs scores were significantly higher in non-responding (NR) patients compared to responding (R) patients. This suggests that FN1 + TAMs may contribute to immunotherapy resistance. With single cell data, the elevated FN1 expression in myeloid cells of the NR group corroborates this finding, indicating that FN1 + TAMs might inhibit effective immune responses necessary for successful immunotherapy. We also developed a comprehensive single-cell immune microenvironment database (PRIMEG) for primary and recurrent gliomas to facilitate further research on the immune landscape of recurrent gliomas. This online database, equipped with a user-friendly interface, provides a valuable resource for advancing the understanding and treatment of recurrent gliomas.

We found that FN1 + TAMs are heterogeneously distributed in gliomas and are primarily associated with hypoxic regions. This association underscores the importance of the metabolic microenvironment in glioma progression and therapeutic resistance. Targeting hypoxia-related pathways and FN1 + TAMs may offer new therapeutic strategies to improve outcomes in glioma patients, particularly in overcoming resistance to immunotherapy. These findings offer novel insights into glioma biology and underscore the potential of FN1 + TAMs as therapeutic targets. Future research should focus on elucidating the underlying mechanisms by which FN1 + TAMs influence glioma progression and immunotherapy outcomes, as well as exploring targeted strategies to modulate their activity.

This study also has some limitations. While correlations were observed, functional validation of the role of FN1 + TAMs in glioma progression and therapy resistance is essential. This includes in vivo studies and experimental manipulation of FN1 + TAM activity. The tumor microenvironment is highly complex, and the interactions between FN1 + TAMs and other cell types or factors were not fully explored. A more comprehensive analysis of these interactions could provide deeper insights. At last, Larger, multicenter studies are necessary to validate FN1 + TAMs as reliable biomarkers for prognosis and therapeutic response. Additionally, clinical trials targeting FN1 + TAMs should be pursued to assess the efficacy of such interventions in improving patient outcomes.

## Conclusion

Our findings identify FN1 + TAMs as key players in glioma recurrence and highlight their potential as biomarkers for prognosis and targets for therapeutic intervention. These insights pave the way for novel treatment paradigms aimed at improving outcomes for patients with recurrent gliomas, particularly in the context of immunotherapy.

## Supplementary Information

Supplementary Material 1: DEGs between high and low RRS TAMs.

Supplementary Material 2: Figure S1. UMAP plot of the developmental trajectory of the TAMs inferred by Slingshot and RNA velocity analysis. Arrows indicate the orientation of the inferred developmental pseudotime trajectory.

## Data Availability

The supplementary material for this article can be found online (For scRNA-seq (10.6084/m9.figshare.22434341; https://brainimmuneatlas.org; ) For spatial transcriptomics (10.6084/m9.figshare.22434341); For RNA-seq and clinical information(GLASS (https://www.synapse.org/glass); CGGA, TCGA and Rembrandt (http://gliovis.bioinfo.cnio.es/); Ivy (https://glioblastoma.alleninstitute.org/); Immunotherapy glioma cohort(GSE79671);Processed Huashan cohorts RNA-seq data was uploaded in synapse: syn61807589)). All processed data and R codes used in this study can be obtained from the corresponding author on reasonable request.

## Abbreviations

SOC: standard of care
TAM: Tumor Associated Macrophages
GAM: Glioma Associated Macrophages
MG: Microglia
BMDM: Bone Marrow-Derived Macrophages
PRIMEG: Atlas of Primary and Recurrent Immune MicroEnvironment of Glioma
CGGA: Chinese Glioma Genome Atlas
RRS: Recurrence-related Signatures
GLASS: Glioma Longitudinal AnalySiS
PC: Pseudopalisading cell
LE: Leading edge
DEG: Differentially expressed genes
ssGSEA: single sample gene set enrichment analysis
SubMap: Subclass mapping
FN1: Fibronectin 1
GSEA: Gene Set Enrichment Analysis
PD/SD: progressive disease/stable disease
PR/CR: partial response/complete response
TME: tumor microenvironment
TIICs: tumor-infiltrating immune cells
