# Supplementary figures and images for: Single-cell RNA sequencing identifies a subtype of FN1 + tumor-associated macrophages associated with glioma recurrence and as a biomarker for immunotherapy
Source: Biomark Res. 2024 Oct 7;12:114. doi: 10.1186/s40364-024-00662-1 (PMC11457430; doi:10.1186/s40364-024-00662-1)

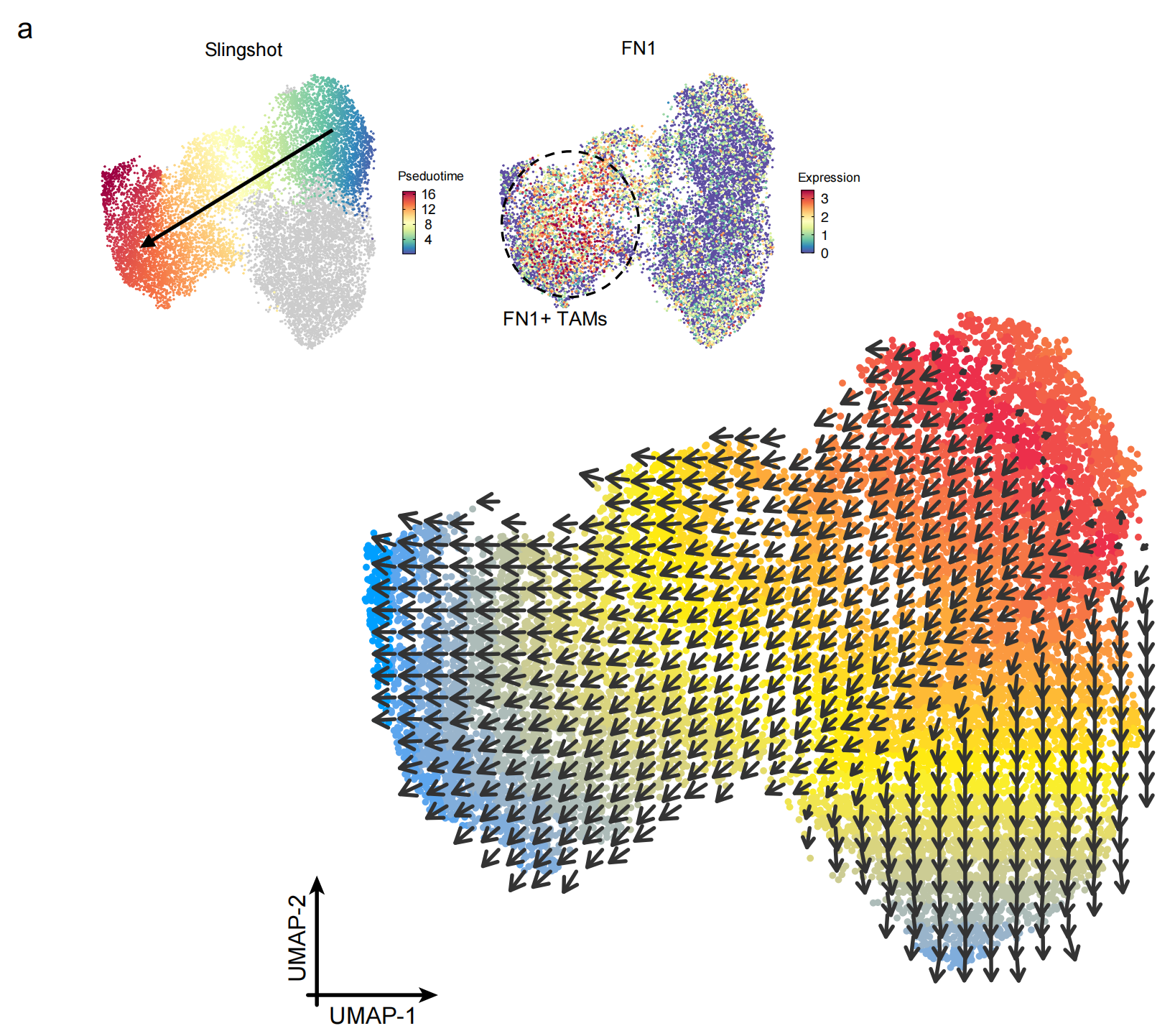

Supplement: Supplementary file 2 — Supplementary Material 2: Figure S1. UMAP plot of the developmental trajectory of the TAMs inferred by Slingshot and RNA velocity analysis. Arrows indicate the orientation of the inferred developmental pseudotime trajectory. [file 40364_2024_662_MOESM2_ESM.tif]
